# CD137 (4-1BB) stimulation leads to metabolic and functional reprogramming of human monocytes/macrophages enhancing their tumoricidal activity

**DOI:** 10.1038/s41375-021-01287-1

**Published:** 2021-05-21

**Authors:** A. Stoll, H. Bruns, M. Fuchs, S. Völkl, F. Nimmerjahn, M. Kunz, M. Peipp, A. Mackensen, D. Mougiakakos

**Affiliations:** 1 Department of Medicine 5 for Hematology and Oncology, Friedrich-Alexander-Universität Erlangen-Nürnberg, Erlangen, Germany; 2 Chair of Medical Informatics, Friedrich-Alexander University (FAU) of Erlangen-Nürnberg, Erlangen, Germany; 3 Functional Genomics and Systems Biology Group, Department of Bioinformatics, University of Würzburg, Würzburg, Germany; 4 Division of Genetics, Department of Biology, Friedrich-Alexander-Universität Erlangen-Nürnberg, Erlangen, Germany; 5 Division of Stem Cell Transplantation and Immunotherapy, Department of Medicine II, Christian-Albrechts-University Kiel, Kiel, Germany; 6 Deutsches Zentrum für Immuntherapie (DZI), Erlangen, Germany

**Keywords:** Translational research, Immunotherapy

## Abstract

Immunotherapies have heralded a new era in the cancer treatment. In addition to checkpoint inhibitors, agonistic antibodies against co-stimulatory immune receptors hold the potential to invoke efficient antitumor immunity. Targeting CD137 has gained momentum based on its ability to drive NK- and T-cell-based responses. CD137-engaging mAbs have already entered clinical trials for different types of tumors showing promising results. Despite the efforts to translate CD137-mediated immunotherapy into clinical practice, little remains known regarding the role of CD137 in human monocytes/macrophages.

We found CD137 being expressed on monocytes of healthy controls and at even higher levels in patients with multiple myeloma or CLL. CD137^HI(GH)^ monocytes displayed a distinct phenotypic, transcriptomic, and metabolic profile. They possessed an increased phagocytic capacity enabling superior antibody-dependent phagocytosis (ADPC) of multiple myeloma and lymphoma cells that were treated with anti-CD38 or anti-CD20 mAbs. Triggering CD137 promoted both metabolic and tumoricidal activity in an extracellular signal-regulated kinase (ERK)-dependent fashion. In addition, we observed a phenotypic, transcriptomic, and functional skewing towards a M1-like phenotype.

Overall, we introduce CD137 as a positive immune checkpoint on human monocytes/macrophages, which can have therapeutic implications especially in view of synergistic effects when combining CD137 agonists with tumor-targeting antibodies.

## Introduction

To date, tumor immunosurveillance and tumor immunoediting are well-established concepts. Moreover, immunotherapy represents one the most appealing anti-cancer approaches [1]. Two key strategies are currently intensely pursued: adoptive transfer of genetically engineered immune cells (e.g., chimeric antigen receptor/CAR T-cells) or application of agents that activate the patients’ immune system (by e.g., blockade of negative immune checkpoints) and thereby re-invigorate intrinsic antitumor immunity. Immune checkpoint receptors are membrane molecules on immune cells, which upon binding to their cognate ligand on tumors or tumor-associated cells can negatively (i.e., inhibitory receptors) or positively (i.e., stimulatory receptors) impact the immune cells’ function. Examples for inhibitory receptors that are successfully targeted in the clinical practice include programmed cell death protein 1 (PD-1) and cytotoxic T-lymphocyte-associated protein 4 [2].

The cell surface glycoprotein CD137, which is also known as 4-1BB, belongs to the group of co-stimulatory immune receptors and is a member of the TNF receptor superfamily. It is preferentially found on activated T-cells and regulatory T-cells (T_Regs_) but also innate immune cells, such as natural killer (NK-) cells, neutrophils, and monocytes can express CD137 [3–5]. Antigen-presenting cells, such as dendritic cells predominantly express, especially in response to stimulatory trigger, its natural ligand CD137-L. The CD137/CD137-L interaction leads to the recruitment of TNF receptor-associated factors 1/2 and the downstream activation of the nuclear factor kappa B transcriptional pathway [6]. In T-cells, CD137 crosslinking delivers a potent co-stimulatory signal as it promotes T-cell proliferation and formation of memory cells, enhances survival, and increases the production of interferon-γ (IFN-γ) and interleukin-2 (IL-2) [7]. Moreover, CD137 signaling has been shown to hold the potential to reprogram tolerogenic T_Regs_ into effector T-cells with antitumor activity [4]. In fact, incorporating the intracellular signaling domain of CD137 fosters clinical activity of CAR T-cells further highlighting its importance for antitumor immunity [8]. Likewise, NK cells respond to CD137 stimulation with increased proliferation, production of IFN-γ, and the ability to perform antibody-dependent cell-mediated cytotoxicity (ADCC) against malignant cells [9].

Consistent with its co-stimulatory function, agonistic monoclonal antibodies (mAbs) against CD137 have elicited effective antitumor immune responses in preclinical models that have been mainly attributed to the activation of T-cells and/or NK cells [10–12]. Actually, CD137-engaging mAbs, such as Urelumab or Utomilumab have already entered clinical trials for different types of tumors (e.g., melanoma or lung cancer) demonstrating their efficacy [13, 14]. However, the role of CD137 signaling in human monocytes/macrophages remains relatively unexplored. In fact, monocyte/macrophage lineage cells accumulate in numerous malignant entities. Tumor-associated monocytes/macrophages (TAMs) regularly display a pro-tumoral M2 phenotype. They do not only provide nurturing signals by e.g., stimulating angiogenesis but also inhibit antitumor immune responses [15]. At the same time, several reports highlight the potential antitumor function of monocytes/macrophages as mediators of antibody-based therapies against cancer, such as anti-CD20 or anti-Her2 mAbs or potentiators of adaptive immune responses [16, 17]. Importantly, intrinsic tumoricidal capacity is retained in the M2-like cells and can be reactivated by disrupting M2-promoting signals leading to the formation of a rather immunoreactive M1 phenotype [18]. Given their central role in tumor development and tumor progression as well as their functional plasticity, monocytes/macrophages represent ideal candidates for therapeutic interventions. However, to fully unleash their tumoricidal capacity, it can be necessary to previously overcome certain immunosuppressive hurdles. Blocking the negative immune checkpoint CD47 or triggering the positive immune checkpoint CD40 has led to an efficient macrophage-mediated tumor regression [19, 20].

In this study, we hypothesized that CD137 might act as a co-stimulatory receptor on human monocytes/macrophages that could hold the potential to enhance their tumor-directed activities. We found CD137 being expressed on circulating monocytes of healthy controls and at even higher levels on cancer patient-derived cells. In fact, CD137^HI(GH)^ monocytes displayed a distinct phenotypic, transcriptomic, and metabolic profile. They possessed an increased phagocytic capacity enabling a superior antibody-dependent phagocytosis of multiple myeloma (MM) and lymphoma cells that were treated with anti-CD38 or anti-CD20 mAbs, respectively. Agonistic triggering of CD137 on myeloid cells promoted both metabolic and tumoricidal activity in an extracellular signal-regulated kinase (ERK)-dependent fashion. In addition, we observed a phenotypic, transcriptomic, and functional skewing towards a M1-like phenotype. Taken together, we identified CD137 as a novel positive immune checkpoint on human monocytes/macrophages, which can have therapeutic implications especially in view of an added therapeutic value when combining CD137 agonists with tumor-targeting mAbs.

## Methods

### Patient material

Peripheral blood mononuclear cells and bone marrow samples from MM patients and healthy donors were collected upon approval by the local ethics committee (Ref. number 3555, 36_12 B, 219_14B, 200_12B) and participants’ written informed consent in accordance with the Declaration of Helsinki.

### Cell lines, antibodies, and primers

Cell lines, antibodies, and primers used are listed in Supplementary Tables 1–3.

### CD137 stimulation

Anti-human CD137 mAbs (Clone 4B4-1, Biolegend) or mouse IgG1, κ isotype control (Biolegend) were diluted to 10 µg/ml in DPBS (ThermoFischer Scientific) and coated to 48-well plate (Corning) at 4 °C overnight. Afterwards, coating solution was removed and MACS- purified monocytes were cultured at 0.5 × 10^6^ /well for 4 days unless otherwise specified. For repolarization experiments, M2 macrophages were cultured instead of monocytes under the same conditions for 3 days.

For the inhibition of ERK1/2 signaling upon CD137 triggering, selective ERK1/2 inhibitors Ravoxertinib or SCH772984 (Selleckchem) were added to culture medium at different concentrations directly after seeding the monocytes.

### Macrophage generation

Monocytes were cultured for 6 days in presence of 50 ng/ml GM-CSF (Berlex, Leukine) or 50 ng/ml M-CSF (R&D Systems) to generate M1- and M2 macrophages, respectively.

### Phagocytosis assays

Target cells were labeled with 0.5 µM CFSE (Invitrogen) in DPBS/ 0.1 %FCS and opsonized with different concentrations of therapeutic antibodies Daratumumab (Janssen, Darzalex®) or Rituximab (Roche, MabThera®). Monocytes were co-cultured with target cells at different effector to target (E:T) ratios in sterile FACS tubes for 4 h. Subsequently, surface staining of cells was performed for FACS analysis.

Phagocytosis of pathogens by healthy donor- or patient-derived monocytes was assessed using pHrodo™ Green E. coli (ThermoFisher Scientific) and analyzed by FACS.

### Tumor cell clearance

Monocytes were co-cultured with CPD-labeled (ThermoFisher Scientific) target cells (E:T = 5:1) in the presence or absence of Daratumumab (10 µg/ml) or Rituximab (10 µg/ml) for 24 h. Monocytes were counterstained with anti-CD11b-FITC. Absolute numbers of surviving target cells were determined by FACS analysis of CD11b^−^/CPD^+^ cells via 123count eBeads (ThermoFisher Scientific).

### Extracellular flux analysis

One day prior to measurements, Seahorse XFe96 culture plates (Agilent Technologies) were coated with Corning™ Cell-Tak Cell and Tissue Adhesive (BD Biosciences). An XFe96 cartridge (Agilent Technologies) was loaded with XF Calibrant solution (Agilent Technologies) and incubated overnight in a CO_2_-free atmosphere. Next, cells were washed in assay-specific medium according to the manufacturer’s recommendations and viability determined using a Muse^®^ Cell Analyzer (Merck Millipore). Viable cells were seeded at a density of 2 × 10^5^ monocytes in 175 µL per well. The ports of the cartridges were loaded with 25 µL each of 80 mM glucose, 9 µM oligomycin, and 1 M 2DG for the glycolysis stress test and 20 µL of 10 µM oligomycin, 22 µL of 15 µM FCCP, and 25 µL of 30 µM antimycin A/rotenone for the mitochondrial stress test. After sensor calibration, assays were run as detailed in the manufacturer’s manual by recording extracellular acidification rate (ECAR) and oxygen consumption rate (OCR). Metabolic parameters were obtained from the XF Wave software (Agilent/Seahorse Biosciences) and calculated using Microsoft Excel.

### Statistics

Statistics were calculated with Graphpad Prism Version 9 (La Jolla, California, USA). Comparisons between groups were performed using the appropriate statistical methods depending on Gaussian distribution and number of groups and variables, i.e., one-way ANOVA with Tukey’s post hoc test, two-way ANOVA with Bonferroni post hoc test, unpaired and paired two-tailed *t*-test, and Mann–Whitney test.

## Results

### CD137 expression level identifies circulating monocytes with a distinct phenotypic and transcriptomic profile in vivo

**Fig. 1:**
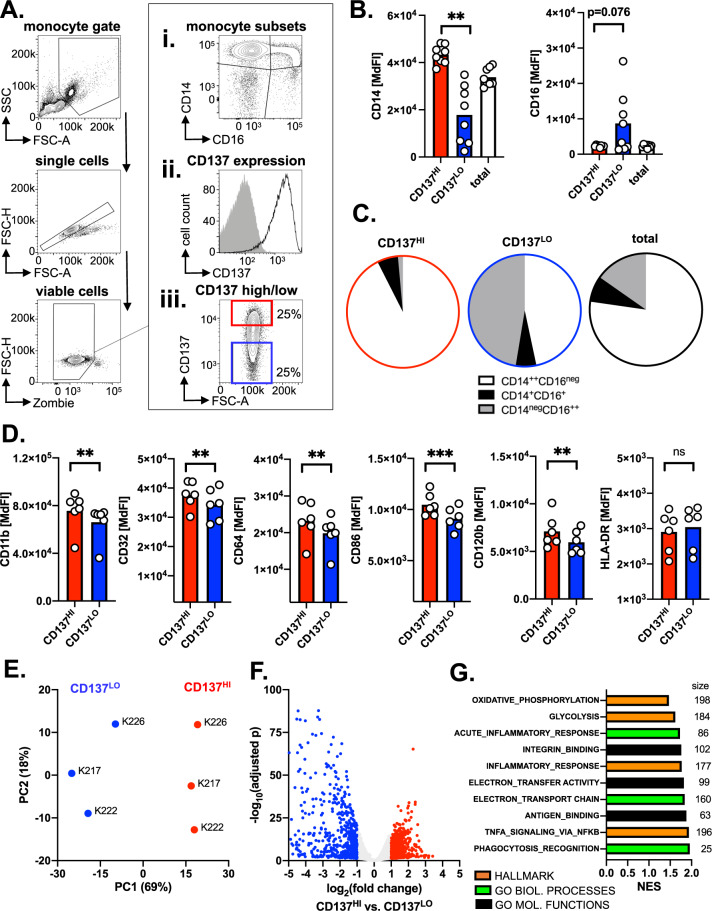
CD137^HI(GH)^ monocytes display and distinct phenotypic and transcriptomic profile. **A** Peripheral blood-derived monocytes were analyzed by flow cytometry (FACS) in terms of CD14 and CD16 expression patterns, CD137 expression, and CD137 expression levels as shown in this representative gating strategy. **B** CD14 and CD16 expression was quantified in CD137^HI^, CD137^LO(W)^, and total monocytes (*n* = 8) by FACS based on the median fluorescence index (MdFI). **C** The proportion of CD14^++^CD16^neg^/classical, CD14^+^CD16^+^/intermediate, and CD14^neg^CD16^++^/non-classical monocytes among CD137^HI^, CD137^LO^, and total monocytes (*n* = 8) was determined by FACS and is shown as a pie chart. **D** Expression of CD11b, CD32, CD64, CD86, CD120b, and HLA-DR was quantified on CD137^HI^ and CD137^LO^ monocytes (*n* = 6) by FACS. **E** CD137^HI^ and CD137^LO^ monocytes (*n* = 3) were purified by FACS-sorting followed by RNA sequencing. The principal component (PC) analysis of differentially expressed genes separates along PC1 according to CD137 expression level. **F** Volcano plot of differentially expressed genes showing genes (each dot represents one gene) with an at least twofold upregulation (red) or downregulation (blue) in CD137^HI^ monocytes, as well as a high number of significantly differentially expressed genes (*q* < 0.1: 5513 genes). **G** Gene set enrichment analysis of differential gene expression between CD137^HI^ and CD137^LO^ monocytes. Graph depicts significantly enriched pathways found in MSigDB hallmark (orange) and gene ontology (green and black) sets. Size of gene set is annotated to the right. Bar length corresponds to normalized enrichment score (NES). *p < 0.05; **p < 0.01; ***p < 0.001.

First, we sought out to assess whether CD137 is expressed on the main subsets of circulating healthy donor-derived monocytes. Subset classification by flow cytometry (FACS) was based on the CD14 and CD16 expression and resulted in three different populations namely, classical (CD14^++^, CD16^neg^), intermediate (CD14^+^, CD16^+^), and non-classical (CD14^neg^, CD16^++^) monocytes (Fig. 1Ai). CD137 was found expressed on all the tested monocytic subtypes (Fig. 1Aii). For the subsequent comparative analyses, we grouped monocytes in accordance to their CD137 expression level into CD137^HI(GH)^ and CD137^LO(W)^ cells (Fig. 1Aiii). In fact, CD137^HI^ cells displayed a significantly higher CD14 expression and a tendency towards lower levels of the Fcγ receptor CD16 (Fig. 1B). Consequently, we found classical monocytes being overrepresented within the CD137^HI^ and non-classical within the CD137^LO^ compartment, respectively (Fig. 1C).

Next, we assessed surface molecules on CD137^HI^ and CD137^LO^ monocytes that have been reported as differentially expressed on classical versus non-classical subsets including CD11b (classical > non-classical), CD32 (classical = non-classical), CD64 (classical > non-classical), CD86 (classical < non-classical), CD120b (classical < non-classical), as well as HLA-DR (classical < non-classical) [21, 22]. In line with the previously reported characteristics of classical and non-classical monocytes, we measured higher levels of CD11b and CD64 on CD137^HI^ cells. Conversely, expression was also superior for CD32, CD86, and CD120b pointing towards an, if at all, only partial overlap between CD137^HI^ and classical monocytes (Fig. 1D).

To confirm distinct features in CD137^HI^ and CD137^LO^ monocytes, we FACS-sorted both populations followed by a RNA-seq analysis (GSE171108). The principal component analysis (PCA) together with the gene expression profiling suggest a separation between both populations (Fig. 1E, F). Furthermore, our gene set enrichment analyses (GSEA) revealed that CD137^HI^ cells are significantly enriched for genes that are amongst others involved in metabolism, inflammation, and phagocytosis (Fig. 1G). When comparing our transcriptome profiles with a publicly available RNA-seq dataset (GSE107011), the notion that CD137^HI^ and CD137^LO^ overlap with classical and non-classical monocytes respectively only partially is further corroborated (Supplementary Fig. 1) [23].

### CD137^HI^ monocytes possess a superior metabolic armamentarium and a proglycolytic phenotype

**Fig. 2:**
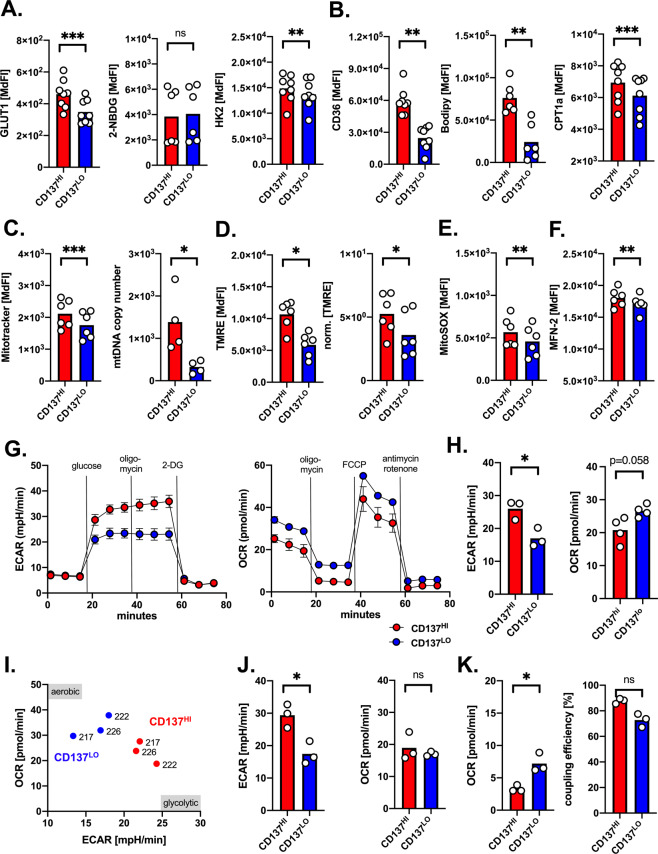
CD137^HI^ monocytes possess a superior metabolic fitness and a proglycolytic phenotype. **A** Expression of glucose transporter GLUT1 (*n* = 8), uptake of glucose analog 6-NBDG (*n* = 6), and glycolytic pacemaker enzyme hexokinase 2 (HK2) level (*n* = 8) were determined by FACS in CD137^HI^ and CD137^LO^ monocytes. **B** Expression of fatty acid transporter CD36 (*n* = 8), uptake of fatty acids linked to the fluorophore BODIPY™ FL C16 staining (*n* = 6), and level of the key enzyme shuttling fatty acids into the mitochondria carnitine palmitoyltransferase I (CPT1) (*n* = 8) were determined by FACS in CD137^HI^ and CD137^LO^ monocytes. **C** The mitochondrial mass in CD137^HI^ and CD137^LO^ monocytes was determined by FACS using the mitochondrial dye MitoTracker (*n* = 6) or by measuring the relative (to nuclear DNA) mitochondrial DNA (mtDNA) copy number (*n* = 4). **D** Mitochondrial membrane potential was assessed in CD137^HI^ and CD137^LO^ monocytes (*n* = 6) by FACS using the potentiometric dye tetramethylrhodamine ethyl ester (TMRE). The right panel shows the TMRE values normalized for cell size. **E** Mitochondrial-specific production of reactive oxygen species (MitoSOX) (*n* = 5) and **F** expression of mitofusin-2 (MFN-2) (*n* = 6), which regulates mitochondrial fusion were determined by FACS in CD137^HI^ and CD137^LO^ monocytes. **G** Graphs display metabolic flux analyses of CD137^HI^ and CD137^LO^ monocytes (*n* = 3). Extracellular acidification rate (ECAR, left panel), was measured as surrogate for aerobic glycolysis under basal conditions, in response to glucose (=basal glycolysis), and upon blocking mitochondrial ATP generation by oligomycin (=maximum glycolysis). 2-DG inhibits glycolysis and blunts ECAR activity. Oxygen consumption (OCR, right panel) was measured as marker for mitochondrial oxidative phosphorylation under basal conditions and in response to the indicated mitochondrial inhibitors. Changes after oligomycin, FCCP, and Antimycin/Rotenone are indicative for respiration linked to ATP production, and maximal respiratory capacity. **H** Basal glycolysis (left panel) and basal respiration (right) panel were determined for CD137^HI^ and CD137^LO^ monocytes (*n* = 3). **I** Basal OCR/ECAR ratio is plotted for individual CD137^HI^ and CD137^LO^ monocytes. **J** Glycolytic capacity (left panel) and spared respiratory capacity (right panel) were determined for CD137^HI^ and CD137^LO^ monocytes (*n* = 3). **K** Proton leak (left panel) and coupling efficacy (right panel) were determined for CD137^HI^ and CD137^LO^ monocytes (*n* = 3). *p < 0.05; **p < 0.01; ***p < 0.001.

Metabolic competence is a prerequisite for monocytes/macrophages to efficiently carry out their antitumoral functions [24]. Our transcriptome analysis revealed an enhanced expression of molecules involved in both glycolysis and oxidative phosphorylation for the CD137^HI^ subset (Fig. 1G). This would fit the observation that CD137 signaling activates glycolysis and fatty acid metabolism in CD8^+^ T-cells [25]. In line with our mRNA profiles, we found higher levels of the glucose transporter 1 (GLUT1) and the glycolytic pacemaker enzyme hexokinase 2 (HK2) in the CD137^HI^ cells. Uptake of the fluorescently labeled glucose analog 6-NBDG was similar in both CD137 groups (Fig. 2A). In addition, the fatty acid transporter CD36, uptake of BODIPY-linked fatty acids, and carnitine O-palmitoyltransferase 1 (CPT1a) the key molecule shuttling long-chain fatty acids into the mitochondrion to be oxidized were all elevated in the CD137^HI^ subset (Fig. 2B).

Next, we concentrated on the mitochondria as one of the cells’ central hubs that integrates bioenergetics and immune responses. Assessment of mitochondrial mass and mitochondrial DNA revealed an increased mitochondrial biogenesis in CD137^HI^ cells (Fig. 2C). The mitochondrial membrane potential (Δψm) has been shown to act as a reliable marker for mitochondrial activity that powers ATP energy formation [26]. CD137^HI^ cells displayed a higher Δψm, which was accompanied by increased mitochondrial reactive oxygen species (MitoSOX) (Fig. 2D, E) and which can be observed during M1 polarization [27]. Mitochondria represent highly dynamic organelles that continuously shift between fusion and fission in response to intrinsic and extrinsic stimuli. Mitofusin-2 (MFN-2) plays a key role for fusion and is also involved in promoting Δψm, mitochondrial ROS production as well as proinflammatory activation, cytokine production, and phagocytosis [28]. In fact, MFN-2 was significantly higher in the CD137^HI^ population (Fig. 2F).

In order to complete our metabolic mapping, we performed metabolic flux analyses of FACS-sorted CD137^HI^ and CD137^LO^ monocytes. ECAR is indicative for aerobic glycolysis and OCR for oxidative phosphorylation (OXPHOS) (Fig. 2G). Basal glycolysis was significantly elevated in CD137^HI^ cells and we observed a strong trend towards a reduced respiratory rate (Fig. 2H). Consequently, the OCR/ECAR ratio in CD137^HI^ cells was skewed towards ECAR resulting in an overall proglycolytic phenotype (Fig. 2I), which is similarly described for monocytes and macrophages following proinflammatory activation [29]. When evaluating the cells’ bioenergetic reserve capacities that co-determine their ability to adapt to cellular stress, we noticed a superior glycolytic reserve in CD137^HI^ cells and a relatively comparable spare respiratory capacity (Fig. 2J). In accordance to the higher Δψm, we observed a reduced proton leak and a trend towards better coupling efficiency in CD137^HI^ cells (Fig. 2K).

### CD137^HI^ monocytes display an enhanced phagocytic activity

**Fig. 3:**
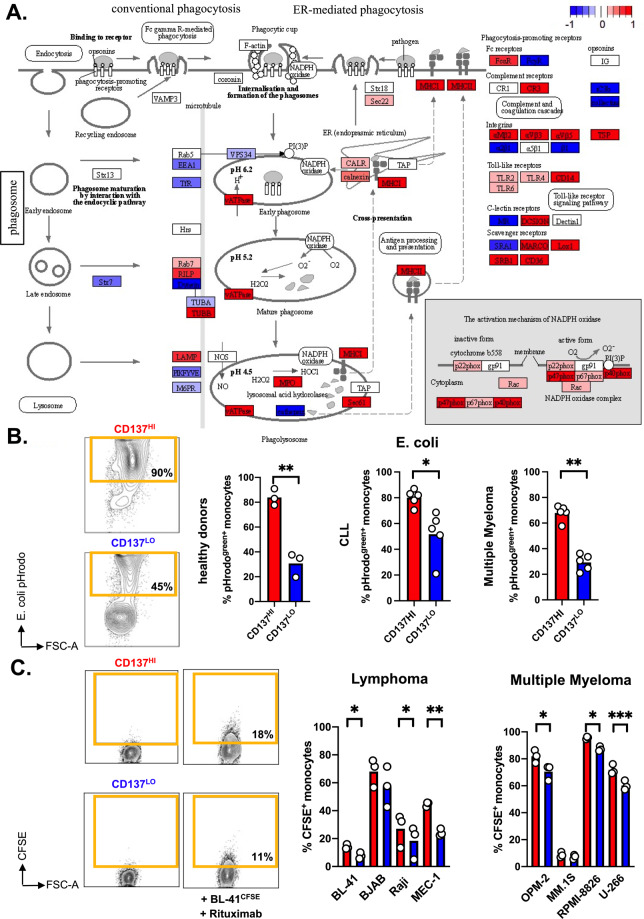
CD137^HI^ monocytes exhibit enhanced phagocytic activity. **A** Differential gene expression of CD137^HI^ and CD137^LO^ monocytes shows enrichment of genes upregulated in CD137^HI^ monocytes (red) in the KEGG Phagosome Reference pathway (map04145). **B** Phagocytosis of pHrodo™-conjugated *E. coli* by CD137^HI^ and CD137^LO^ monocytes from healthy donors (*n* = 3) and patients with chronic lymphocytic leukemia (CLL, *n* = 5) or multiple myeloma (*n* = 5). Left panel shows representative FACS-based analyses of healthy donor-derived monocytes taking up pHrodo™-conjugated *E. coli*. **C** Phagocytosis of CFSE-stained tumor cells in presence of therapeutic antibodies by CD137^HI^ and CD137^LO^ monocytes as analyzed by FACS. The left panel shows representative data for the uptake of the Burkitt lymphoma cell line BL-41 in presence of Rituximab. Phagocytosis by CD137^HI^ and CD137^LO^ monocytes (*n* = 3) of CD20^+^ B-cell-derived non-Hodgkin lymphoma cell lines (in presence of Rituximab) and of CD38^+^ multiple myeloma cell lines (in presence of Daratumumab). *p < 0.05; **p < 0.01; ***p < 0.001.

The RNA-seq analysis of CD137^HI^ *versus* CD137^LO^ monocytes revealed the increased expression of a number of molecules involved in phagocytosis as accordingly mapped in the KEGG “Phagosome” pathway (hsa04145) (Fig. 3A). Phagocytosis plays an important role in the host immune defense as well as in antitumor immunity. In order to validate our gene expression data, we cultured CD137^HI/LO^ monocytes from healthy donors but also patients with chronic lymphocytic leukemia (CLL) or MM in presence of conjugated *E. coli* bioparticles. In this flow cytometry-based assay a positive signal occurs upon particle internalization and acidification, which are activities indicative for phagocytic actions. In line with the RNA-seq data, we found a significantly higher fraction of positive cells among the CD137^HI^ population (Fig. 3B).

Tumor-targeting antibodies are considered to be one of the most successful strategies in cancer therapy. The antigens CD20 and CD38 are expressed on most B-cell-derived malignancies and MM cases respectively, which has been translated into efficient mAb-based therapies [30, 31]. Myeloid cells are abundantly present in the microenvironment of both entities [32, 33]. Therefore, we sought out to evaluate the ability of CD137^HI/LO^ monocytes to clear malignant cell in presence of tumor-targeting mAbs. First, we evaluated several fluorescently labeled CD20^+^ B-cell malignancy-derived cell lines (Burkitt lymphoma: BL-41, BJAB, and Raji, CLL: MEC-1) applying the clinically approved anti-CD20 mAb Rituximab. As anticipated, antibody-dependent cellular phagocytosis (ADCP) was superior in CD137^HI^ cells. Moreover, we made equivalent observations, when using CD38^+^ MM-derived cell lines (OPM-2, MM.1 S, RPMI-8826, U-266) that were treated with the anti-CD38 mAb Daratumumab (Fig. 3C, Supplementary Fig. 2).

### CD137 stimulation promotes metabolic and tumoricidal activity of monocytes

TAMs are components of the tumor microenvironment and often associated with a dismal prognosis. However, reprogramming TAMs represents a promising strategy for positively instrumentalizing them. This approach is based on the plasticity of monocytes/macrophages, whose different phenotypes form a continuum between an antitumoral M1 and a rather pro-tumoral M2 phenotype. In fact, intrinsic tumoricidal capacity is retained in those M2-like TAMs and can be reactivated in preclinical models by disrupting M2-promoting signals or by interfering with immunological checkpoints [18, 34]. Here, we wanted to evaluate, whether CD137 stimulation by agonistic anti-CD137 antibodies holds the potential to bolster the monocytes’ tumoricidal activity, especially in view of a combination with tumor-targeting mAbs [9].

**Fig. 4:**
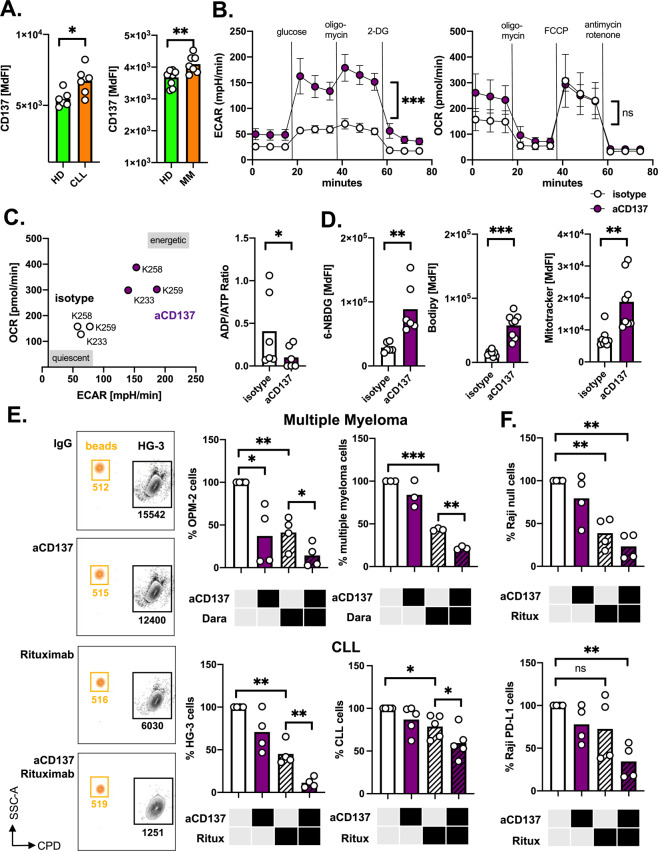
CD137 stimulation on monocytes increases metabolic activity and tumoricidal capacity. **A** CD137 expression was quantified by FACS on healthy donor-derived monocytes (HD) and monocytes from CLL (*n* = 6) and multiple myeloma (*n* = 7) patients. **B** Extracellular flux analyses were performed with monocytes treated with the 4B4-1 CD137-stimulating monoclonal antibody or a control antibody (*n* = 3) for 4 days. ECAR (left panel) and OCR (right panel) levels were measured under baseline conditions and upon injection of the indicated substances. **C** Basal ECAR/OCR ratio (left panel) of control vs. anti-CD137 mAb-treated monocytes (*n* = 3) is plotted showing a shift from quiescent to metabolically more active cells. The right panel displays the ratio of ADP/ATP of control vs. anti-CD137 mAb-treated monocytes (*n* = 6). **D** Glucose uptake assessed by 6-NBDG staining (*n* = 6), fatty acid uptake assessed by BODIPY™ FL C16 staining (*n* = 8), and mitochondrial biomass assessed by MitoTracker staining (*n* = 8) of control vs. anti-CD137 mAb-treated monocytes were analyzed by FACS. **E** Killing of CPD-stained tumor cells in presence or absence of a therapeutic antibody (Dara = Daratumumab, Ritux = Rituximab) by monocytes pretreated with either control or anti-CD137 mAb for 3 days. Left panels show representative FACS plots of surviving lymphoma cells (HG-3) after 18 h of coculture. Counting beads were used for normalization of per sample tumor cell counts. Upper panels showing killing of multiple myeloma cell lines OPM-2 (*n* = 4) and of primary multiple myeloma cells co-cultured with autologous bone marrow-derived macrophages (*n* = 3) and the lower panels show killing of the CLL cell line HG-3 (*n* = 4) and of primary CLL cells by autologous monocytes (*n* = 5). Survival of tumor cells co-cultured with control mAb-treated monocytes without therapeutic antibody are set to 100%. **F** Experimental setup as described in **E** using a PD-L1 overexpressing Raji cell line (lower panel) and the corresponding control Raji null (upper panel) (*n* = 4). Bars indicate the standard error of mean. *p < 0.05; **p < 0.01; ***p < 0.001.

Self-evidently, one prerequisite for such CD137-directed approach is the presence of CD137 on patient-derived monocytes. Similar to findings in T-cells isolated from patients with ovarian cancer or melanoma, CD137 expression levels were elevated on monocytes from CLL and MM patients (Fig. 4A) [35]. Moreover, CD137^HI/LO^ CLL or MM monocytes share similar features as their healthy donor-derived counterparts in terms of phagocytosis (Fig. 3B), phenotypic markers (i.e., CD11b, CD32, CD64, CD86, and CD120b), and their metabolic repertoire (i.e., GLUT1, HK2, CD36, CPT1a, Mitotracker, and TMRE) (Supplementary Fig. 3). Next, we stimulated CD137 on healthy donor-derived monocytes by using an agonistic anti-CD137 mAb (clone 4B4-1) and tested for changes of the metabolic phenotype. Metabolic flux analyses revealed an upregulation of both glycolytic (e.g., basal glycolysis and glycolytic reserve) and OXPHOS (e.g., basal respiration) parameters (Fig. 4B, Supplementary Fig. 4). Overall, the monocytes’ bioenergetics shifted to a more metabolically active state leading also to an expected skewing of the ADP-to-ATP ratio towards the latter (Fig. 4C). In line with this enhanced metabolic turnover, uptake of the bioenergetic substrates, such as glucose and free fatty acids as well as mitochondrial biogenesis were found enhanced upon CD137 triggering (Fig. 4D).

Next, we assessed the impact of CD137 activation on the monocytes’ tumoricidal potency. Engaging CD137, resulted in an amplified elimination of the MM cell line OPM-2 presumably through mechanisms of ADCP and ADCC. This was also true when co-applying Daratumumab. Similar phenomena were documented in experimental setups using primary MM-cells and autologous bone marrow-derived macrophages or when testing the B-cell malignancy-derived cell lines HG-3 (CLL) and Raji (Burkitt lymphoma) or primary CLL cells in presence of Rituximab (Fig. 4E, Supplementary Fig. 5A, B). It is well-established that the interaction of PD-L1 (on tumor cells) with PD-1 on monocytes and macrophages drives their anergy [24, 36]. Consequently, Rituximab did not significantly boost tumoricidality, when using PD-L1-overexpressing Raji cells as a target. However, combined treatment with Rituximab and CD137 activation efficiently antagonized the negative impact of PD-L1 overexpression leading to a significantly improved elimination of Raji cells (Fig. 4F, Supplementary Fig. 5C).

### Monocytes/macrophages acquire M1-like features upon stimulation of CD137

**Fig. 5:**
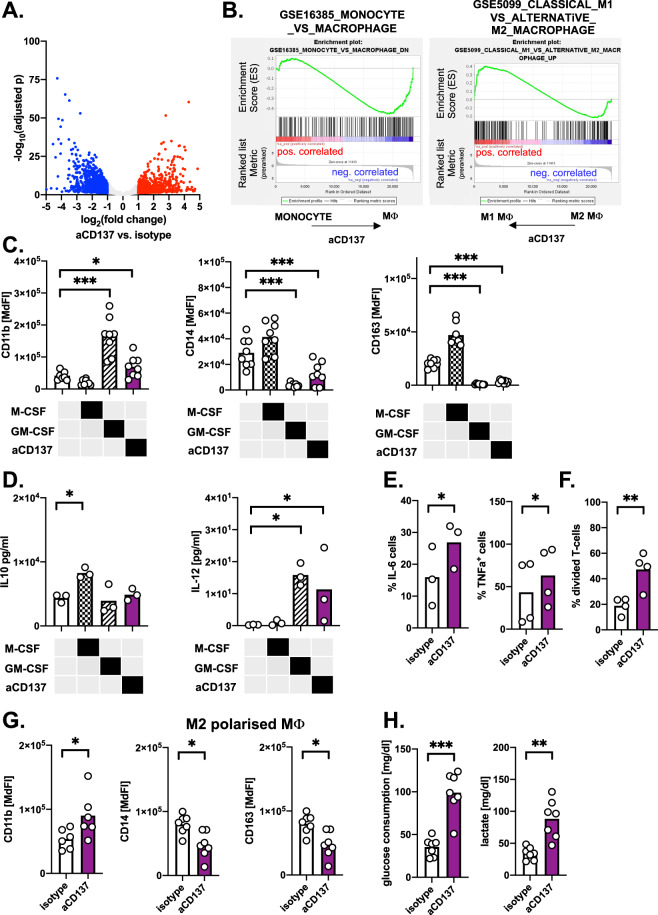
CD137 stimulation on monocytes induces differentiation of macrophages with M1-like features and increased proinflammatory properties. **A** Volcano plot of differentially expressed genes showing genes (each dot represents one gene) with at least twofold upregulation (red) or downregulation (blue) in anti-CD137 mAb-treated monocytes, as well as a high number of significantly differentially expressed genes (*q* < 0.1: 7309 genes). **B** Gene set enrichment analysis of differential gene expression reveals negative enrichment of genes associated with undifferentiated monocytes (left panel, NES = −1.3402828, *p* = 0.03, *q* = 0.12) and significant enrichment of M1 polarization-associated genes (right panel, NES = 1.2730565, *p* = 0.027 *q* = 0.18) in anti-CD137 mAb-treated monocytes. **C** Expression of CD11b, CD14, and CD163 was quantified by FACS on monocytes treated for 6 days with a control antibody, M-CSF (50 ng/ml), GM-CSF (50 ng/ml), or agonistic anti-CD137 mAbs (*n* = 8). **D** IL-10 and IL-12 production was measured by ELISA in the supernatants of monocytes treated for 5 days with a control antibody, M-CSF (50 ng/ml), GM-CSF (50 ng/ml), or agonistic anti-CD137 mAbs and subsequently stimulated with 100 ng/ml LPS for 24 h (*n* = 3). **E** IL-6 (*n* = 3) and TNF-α (*n* = 4) positive (^+^) monocytes were determined by FACS among cells treated for 4 days with a control antibody or agonistic anti-CD137 mAbs and subsequently stimulated with 100 ng/ml LPS for 24 h. **F** Proliferation of T-cells in presence of activation beads (anti-CD2, -CD3, and -CD28 bead-coupled antibodies) co-cultured with HD monocytes (ratio 1:1) pretreated with either control antibody or an anti-CD137 mAb for 4 days (*n* = 5). **G** Expression of CD11b, CD14, and CD163 was quantified by FACS on monocytes (*n* = 7) treated for 4 days with M-CSF followed by a 3-day treatment with a control or anti-CD137 mAb. **H** Glucose consumption and lactate production were determined in supernatants of monocytes treated as in **H** using a HITADO SuperGL. *p < 0.05; **p < 0.01; ***p < 0.001; NES normalized enrichment score.

We performed RNA-seq analyses of monocytes treated with or without activating anti-CD137 antibodies (GSE171109). The unsupervised PCA separated treated from untreated monocytes, thus supporting the notion that triggering CD137 initiates a rather specific gene expression pattern (Supplementary Fig. 6A). Subjecting the genes upregulated in response to CD137 activation to GSEA, demonstrated an enrichment of genes that are linked to the generation of monocyte-derived macrophages, M1 polarization, inflammatory signaling, metabolism, and phagocytosis (Fig. 5A, B, Supplementary Fig. 6B).

Next, monocytes were cultured under M1 and M2 macrophage-polarizing conditions using GM-CSF and M-CSF respectively, or in presence of CD137-stimulating antibodies. Analyzing the surface expression of CD11b, CD14, and CD163 demonstrated that CD137 activation might mimic polarization towards a (rather tumoricidal) M1 phenotype that is characterized by upregulation of CD11b and downregulation of CD14 and CD163 (Fig. 5C) [37]. Equivalent effects were observed, when treating CLL or MM patient-derived monocytes with anti-CD137 mAbs (Supplementary Fig. 7). Moreover, we assessed production of the prototypical cytokines IL-10 (for M2) and IL-12 (for M1) and again observed that both GM-CSF and CD137 activation promoted IL-12 levels (Fig. 5D). We detected an increased production of the proinflammatory cytokines IL-6 and TNF-α, which was accompanied by the significant promotion of T-cell proliferation, when T-cells were co-cultured with previously CD137-stimulated monocytes (Fig. 5E, F).

In order to evaluate, whether CD137 signaling holds the potential to repolarize (already) differentiated M2 macrophages, M-CSF-generated M2-like macrophages were treated with activating anti-CD137 mAbs. Triggering CD137 promoted CD11b expression and suppressed CD14 and CD163 on M2 macrophages, thereby mimicking the effects of GM-CSF that shifts macrophages towards an M1-like phenotype (Fig. 5G). Furthermore, we noticed enhanced glucose consumption and lactic acid release suggesting a proglycolytic shift, which is typically seen in classically activated M1 macrophages (Fig. 5H) [38].

### CD137-mediated effects depend on ERK signaling

**Fig. 6:**
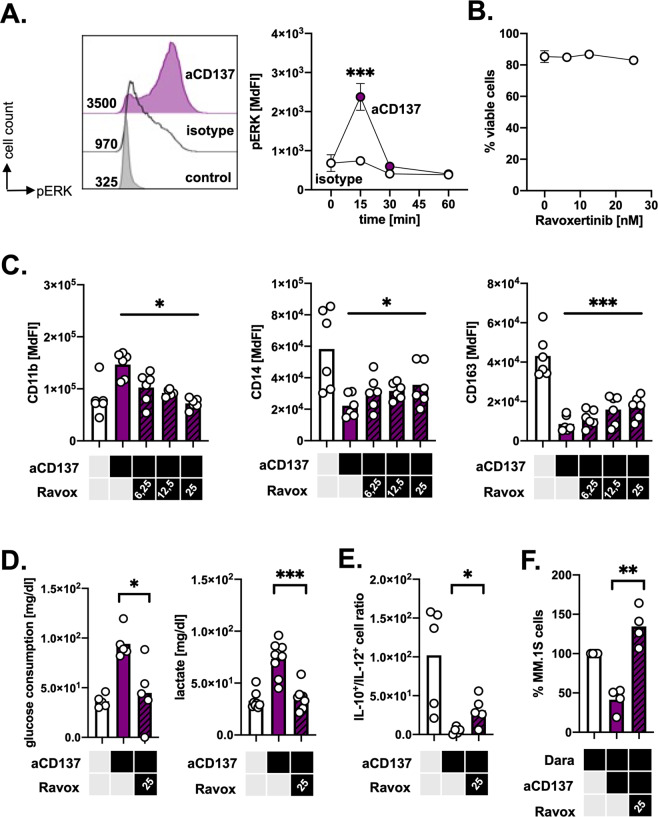
CD137 activation-mediated immunometabolic effects depend on ERK1/2 signaling. **A** pERK staining in human monocytes upon treatment with either 4B4-1 an activating anti-CD137 mAb or an isotype control as determined by FACS. Left panel shows representative pERK staining after 15 min of activation. Right panel shows pERK levels at indicated timepoints after activation. (*n* = 4). **B** Viability of monocytes cultured with indicated concentrations of the ERK inhibitor Ravoxertinib for 4 days as determined by FACS (*n* = 6). **C** Expression of CD11b, CD14, and CD163 was determined on monocytes treated with either control antibody, anti-CD137 mAb or a combination of anti-CD137 mAb and the indicated concentrations (in nM) of Ravoxertinib for 4 days (*n* = 6). **D** Glucose consumption and lactate production were determined in the supernatants of monocytes treated with either control antibody, anti-CD137 mAb or a combination of anti-CD137 mAb and 25 nM of Ravoxertinib for 4 days (*n* = 6). **E** The ratio of IL-10^+^/IL-12^+^ monocytes as determined by an IL-10/12-secretion assay using FACS. Monocytes (*n* = 5) were treated with either control antibody, anti-CD137 mAb or a combination of anti-CD137 mAb and 25 nM of Ravoxertinib for 4 days and subsequently activated with 1 µg/µl LPS and 2 µg/µl Resiquimod for 20 h before secretion assay was performed. **F** Elimination of MM.1S tumor cells in the presence of therapeutic antibody Daratumumab by monocytes pretreated with either control antibody, anti-CD137 mAb or a combination of anti-CD137 mAb and 25 nM of Ravoxertinib for 3 days. Survival was analyzed after 18 h of coculture. Survival rate of MM.1S cells in coculture with isotype-treated monocytes is set to 100%. *p < 0.05; **p < 0.01; ***p < 0.001.

ERK controls numerous molecules involved in translation regulation. Data from previous studies in T-cells suggest that ERK is involved in the downstream signaling of CD137 [39]. Moreover, ERK activation has been shown to convert suppressive macrophages towards a tumoricidal phenotype [40]. In line with the findings in T-cells, we observed ERK phosphorylation in monocytes upon triggering CD137 (Fig. 6A). For the following functional assessment of ERK, we used the pharmacological ERK inhibitor Ravoxertinib in non-cytotoxic dosages (Fig. 6B). Ravoxertinib significantly mitigated in a dose-dependent manner the CD137-mediated effects on the monocytes’ cell surface expression of CD11b, CD14, and CD163 (Fig. 6C). In addition, it prevented their metabolic switch towards aerobic glycolysis as well as the promotion of IL-12 production (Fig. 6D, E). Taking into consideration potential off-target effects of Ravoxertinib, we tested SCH772984, a second extremely selective ERK inhibitor, and confirmed the impact of ERK inhibition on the phenotypic and metabolic transition of monocytes treated with anti-CD137 mAbs (Supplementary Fig. 8). Finally, ERK inhibition fully abolished our previously noticed added tumoricidal value of combining CD137 activation with mAbs against tumor antigens. Engaging CD137 in presence of Ravoxertinib did not improve anymore clearance of the CD38^+^ MM cell line MM.1S treated with the anti-CD38 mAb Daratumumab (Fig. 6F).

## Discussion

Immunotherapeutic concepts have heralded a new era of cancer treatment leading to long-term survival and even cure [1]. Despite this tremendous success, primary resistance and/or acquired resistance during therapy is regularly observed in cancer patients [41]. Poor responses towards immunotherapy are partly attributed to the complex and dynamic nature of the tumor microenvironment. Myeloid cells, such as TAMs or myeloid-derived suppressor cells represent major components of the tumor microenvironment and their abundance can be associated with poor prognosis (e.g., in MM or in aggressive lymphoma) [32, 33]. However, owed to their plasticity, intrinsic tumoricidal capacity is retained in TAMs and efficiently reactivated by disrupting tolerance-promoting signals or by interfering with negative immunological checkpoints, such as CD47 or PD-L1 [18, 19, 36]. In addition to blocking inhibitory signals, engaging stimulatory receptors on immune cells, such as CD137, which has been mainly studied in T and NK cells, represent an area of intensive research [5].

Here, we demonstrate that CD137 is expressed on circulating monocytes, which is in line with previous observations [42]. Moreover, we show that CD137^HI^ monocytes are characterized by a distinct phenotypic and transcriptomic profile. Interestingly, we observed an overrepresentation of classical monocytes within the CD137^HI^ monocyte population accompanied by a functional and phenotypical overlap between both. In line with previous findings in classical monocytes, CD137^HI^ monocytes also exhibited an enhanced carbohydrate metabolism and phagocytic capacity. Furthermore, in transcriptome analyses, CD137^HI^ monocytes were predicted to be of proinflammatory phenotype, which is a known feature of classical monocytes [43, 44]. In fact, these features also hold true for M1 polarized monocytes. Yet, we could not unequivocally assign CD137^HI^ cells to a M1 or an M2 phenotype. This is in line with the general reassessment of the M1/M2 paradigm pointing towards the co-existence of M1 and M2 signatures with the resultant mixed phenotypes rather depending on the balance of activatory and inhibitory signals [45]. Genes that were involved in bioenergetics, proinflammatory activity, and phagocytosis were found enriched in the CD137^HI^ population. Subsequent metabolic analyses revealed a proglycolytic phenotype and an increased glycolytic reserve in CD137^HI^ as compared to CD137^LO^ monocytes. Enhanced glycolysis enables them to meet their energetic demands for carrying out their effector functions, such as phagocytosis and cytokine production [46]. A proglycolytic shift has been shown to be paramount for the monocytes’ and macrophages’ proinflammatory activation [47]. However, it needs to be pointed out that complex stimuli elicit a more complex metabolic response leading to parallel stimulation of both glycolysis and OXPHOS [48]. In line with their metabolic and transcriptomic profile, CD137^HI^ monocytes displayed a superior phagocytic capacity in terms of bacterial pathogens as well as lymphoma or MM cell lines that were pretreated with anti-CD20 and anti-CD38 mAbs [24]. Equivalent findings were recently described in hepatocellular carcinoma patient-derived T-cells, with the CD137^HI^ CD8^+^ subset displaying a superior antitumor reactivity and expressing, amongst others, higher IFN-γ levels [49]. Similar to T-cells retrieved from patients with ovarian cancer or melanoma, we detected elevated CD137 expression levels on circulating monocytes from CLL and MM patients [35].

Using activating antibodies against CD137 on monocytes led to an upregulation of both glycolysis and OXPHOS, which is also observed in CD8^+^ T-cells treated with anti-CD137 mAbs [25]. Overall increased metabolic fitness was accompanied by the monocytes’ superior ability to ingest MM and lymphoma cell lines that are co-treated with anti-CD20 and anti-CD38 antibodies, respectively. These findings are in line with data from preclinical lymphoma and MM models in which CD137 stimulation enhanced the anti-lymphoma and -myeloma activity of anti-CD20 and anti-CD38 antibodies [9, 50, 51]. Remarkably, PD-L1-mediated monocyte inhibition could be overridden by triggering CD137, which could contribute to the synergistic antitumor activity reported in patients with advanced solid tumors that were treated with a combination of PD-1 blockade and CD137 stimulation [24, 52].

Being fully aware of the limitations of the M1/M2 paradigm, we used an in vitro polarization model, in which we generated (antitumoral) M1 and (pro-tumoral) M2 macrophages [45]. Treatment with agonistic CD137 mAbs mimicked M1-polarizing conditions. The notion that CD137 stimulation might hold the potential to skew the M1/M2 balance within the tumor microenvironment towards a proinflammatory M1 profile was further supported by the finding that CD137 stimulation resets phenotypic and metabolic M2-features in already differentiated M2-like macrophages. Moreover, TAMs retrieved from MM patient-derived bone marrow exhibited increased antitumoral activity upon CD137 activation. Accordingly, CTX-471 a novel CD137 agonist with remarkable efficacy in preclinical models of large tumors resulted in a repolarization of tumor microenvironment-resident macrophages towards an M1 phenotype [53].

CD137 stimulation induces rapid downstream activation of the ERK pathway in T-cells, which we also observed in monocytes [39]. In fact, ERK signaling has been previously linked to the conversion of suppressive macrophages towards a pro-immunogenic phenotype [40]. Consequently, blocking ERK signaling in monocytes abolished the immunometabolic effects triggered by CD137 stimulation including enhanced aerobic glycolysis, IL-12 production, and ADCP.

Taken together, we describe an as yet unknown role of CD137 for monocytes/macrophages. Nowadays, targeting CD137 has gained momentum in preclinical and clinical studies based on its ability to drive NK- and T-cell-based responses. Here, we initially reveal that CD137^HI^ monocytes hold a superior tumoricidal potential. Next, we report that activating CD137 on monocytes/macrophages triggers their functional reprogramming towards a metabolically more active, M1-like phenotype with greater antitumor potential, thereby explaining observations from clinical trials and preclinical models that the combination of CD137 agonists and therapeutic antibodies can be effective in malignant diseases. Our findings help to better understand the effects of CD137 activation on the tumor microenvironment, which is critical for its optimal application, selection of biological read-outs in studies, and the choice of therapeutic combination partners.

## Supplementary information

Supplemental Information
