# Supplementary material for: CD137 (4-1BB) stimulation leads to metabolic and functional reprogramming of human monocytes/macrophages enhancing their tumoricidal activity
Source: Leukemia. 2021 May 21;35(12):3482–96. doi: 10.1038/s41375-021-01287-1 (PMC8632678; doi:10.1038/s41375-021-01287-1)
Supplement: Supplementary file 1 — Supplemental Information [file 41375_2021_1287_MOESM1_ESM.pdf]

Supplemental Information

Supplemental Material and Methods

Cell isolation

Mononuclear cells were isolated using Ficoll-Paque (GE Healthcare, Piscataway Township, NJ). Monocytes and T-cells were isolated using the Pan Monocyte Isolation Kit (Miltenyi Biotec) and Pan T-cell Isolation Kit (Miltenyi Biotec), respectively, according to the manufacturer's instructions. Cells of at least 90% purity were used for experiments.

In some cases, adherence isolation for monocytes was performed. PBMCs were seeded at 6 x 10^6 per well in a 12-well plate (Greiner bio-one) in IMDM (ThermoFischer Scientific) supplemented with 10% FCS (PAN BioTech) and 40 U/ mL Penicillin-Streptomycin (ThermoFisher Scientific) and left to adhere for 1 h at 37°C. Afterwards, non-adherent cells were removed.

Cell culture

Cells were cultured at a density of 1 x 10^6/ ml in RPMI1640 (ThermoFisher Scientific) supplemented with 2 mM L-Glutamine (Sigma-Aldrich), 10% FCS (PAN BioTech), and 40 U/ mL Penicillin-Streptomycin (ThermoFisher Scientific) at 37°C and 5% CO_2 atmosphere if not stated otherwise. Mycoplasma contamination was regularly assessed using the Venor® GeM Classic Mycoplasma Detection kit (Minerva biolabs, Berlin, Germany) according to the manufacturer's instructions. Regular cell authentication of cell lines was not performed.

Flow cytometry (FACS)

All surface and intracellular staining included a dead cell exclusion using Zombie AquaDye (Biolegend). Cells were washed in PBS and blocked with FcR Blocking Reagent, human

(Miltenyi Biotec) or human immunoglobulins (Grifols, Gamunex®) prior to the staining. Intracellular staining was performed with a Fixation/Permeabilization Solution Kit (BD Biosciences). For intracellular detection of phosphorylated proteins Phosflow™ Fix Buffer I and Phosflow™ Perm Buffer III (BD Biosciences) were used.

Glucose uptake was analyzed using the fluorescent glucose analog 6-NBDG (ThermoFisher Scientific). Fatty acid uptake was semi-quantified by use of the fluorescently labeled long chain fatty acid, BODIPY™ FL C16 (ThermoFisher Scientific). Mitochondrial superoxide production was assessed by MitoSOX™ Red (Thermo Fisher Scientific) staining according to the manufacturer's recommendations. To analyze the mitochondrial biomass and fitness MitoTracker® Green (ThermoFisher Scientific) and Tetramethylrhodamine ethyl ester (TMRE, Cayman Chemicals) were used respectively.

ADP/ATP Assay

ADP/ATP ratio of in vitro stimulated monocytes was assessed applying the ADP/ATP Ratio Assay Kit (Sigma-Aldrich) with 10^4 cells/ sample as per manufacturer's instructions.

Glucose and lactic acid

Glucose and lactic acid in cell culture supernatants were measured with a HITADO SuperGL compact system according to the manufacturer's instructions.

T-cell proliferation assay

In vitro stimulated monocytes were subsequently co-cultured with autologous VPD-labeled T-cells in different ratios. T-cells were stimulated for five days using anti-CD2, -CD3 and -CD28 bead-coupled antibodies (Miltenyi Biotec), and proliferation was assessed by FACS.

Cell sorting

Purified monocytes were stained with CD14-APC, CD16-BV421, and CD137-PE/Cy7. Cell sorting was carried out on a FACS Aria II (BD Biosciences).

IL-10 and IL-12 ELISA and secretion assay

Detection of IL-10 and IL-12 in supernatants of monocytes treated with a control antibody, M-CSF (50 ng/ml), GM-CSF (50 ng/ml), or agonistic anti-CD137 mAbs for five days, followed by activation with LPS (100 ng/ml) for 24 hours was performed by Human IL-10 ELISA MAX™ Deluxe (Biolegend) and Human IL-12 ELISA MAX™ Deluxe (Biolegend).
For detection of IL10- and IL12-producing cells after four days of CD137 stimulation, monocytes were harvested and reseeded at 0.4 x 10^6 /well in a 24 well plate. Next, cells were either activated with 1 µg/µl LPS and 2 µg/µl Resiquimod for 20 h or left untreated. Subsequently, two-color cytokine secretion assay was performed using IL-10 detection Kit (Miltenyi Biotec) and IL-12 detection Kit (Miltenyi Biotec).

DNA/RNA isolation, quantitative real-time PCR and RNA-sequencing

DNA and RNA were isolated from monocytes using the innuPREP DNA/RNA Mini Kit (analytic Jena). DNA/RNA content was quantified by means of a NanoDrop™ (Thermo Fisher Scientific). For qPCR, RNA was reverse transcribed into cDNA using the SuperScript™ II Reverse Transcriptase (Thermo Fisher Scientific). Gene expression was quantified using the Rotor-Gene SYBR® Green PCR Kit (Qiagen, 204076) and dedicated primers on a Rotor-Gene Q (Qiagen) employing the 2-standard method.

Samples designated for RNA-sequencing (RNAseq) were further processed for removal of residual genomic DNA using the RNase free DNase set (Qiagen).

For RNAseq of *ex vivo* sorted monocytes "INVIEW Transcriptome" (Eurofins/GATC Biotech) product was purchased and RNAseq was performed by Eurofins/GATC Biotech. A strand-specific cDNA library was prepared and sequencing was performed on a HiSeq4000 with >30 x 10^6 50bp single end reads.

RNA-sequencing of *in vitro* stimulated monocytes was performed by the next generation sequencing core unit of the FAU Erlangen-Nürnberg. The cDNA library was prepared using TruSeq Stranded mRNA Kit (Illumina). Libraries were sequenced on a HiSeq2500 sequencer (Illumina) with 95bp single end reads.

All samples were processed as follows: First, reads were mapped against the human genome (GENCODE GRCh38.p13) using STAR v2.7.3a. Second, all non-multimapping and non-multi-overlapping reads mapping to exons were counted using featureCounts v2.0.0. Differential gene expression analysis was performed in R v3.6.1 using DESeq2 v1.26.0. The "Approximate Posterior Estimation for GLM" model was used to assess differential expression for each gene (apeglm v1.8).

Gene set enrichment analysis v4.0.3 (GSEA, http://broadinstitute.org/gsea) was performed to determine enriched gene sets in *ex vivo* CD137$^{HI/LO}$ and *in vitro* CD137-triggered monocytes. Preranked GSEA was performed on gene set collections from MSigDB v7.1 **(**https://www.gsea-msigdb.org/gsea/msigdb/index.jsp**)** using Log_2 fold-change, as determined by DESeq2, as ranking metric. Collections used were: HALLMARK, KEGG, and GO as well as selected gene sets for monocyte/macrophage differentiation.

Supplemental Tables

Supplemental Table 1: Cell lines

**Celllines**	**Provider**	**Order No.**
**Myeloma**		
OPM-2	DSMZ	ACC50
RPMI-8226	DSMZ	ACC402
U-226	DSMZ	ACC9
MM.1S	ATCC	CRL-2974
**CLL**		
EHEB	DSMZ	ACC67
Mec-1	DSMZ	ACC497
HG-3	DSMZ	ACC765
**Burkitt**		
Raji	Kind gift from the Malmberg group at the Oslo university hospital	
BL-41		
BJAB		
Raji-Null	Invivogen	raji-null
Raji-hPD-L1	Invivogen	raji-hpdl1

Supplemental Table 2. Antibodies for flow cytometry

**Antibodies**	**Fluoro-chrome**	**Clone**	**Manufacturer**	**Order No.**
**CPT1a**	AF488	8F6AE9	Abcam	ab171449
**HK2**	-	EPR20839	Abcam	ab209847
**Goat Anti-Rabbit IgG H&L**	AF647	polyclonal	Abcam	ab150079
**HLA-DR**	FITC	G46-6	BD	555811
**CD11b**	APC	ICRF44	Biolegend	301310
**CD11b**	PE	ICRF44	Biolegend	301305

**CD11b**	FITC	ICRF44	Biolegend	301330
**CD14**	PerCP	HCD 14	Biolegend	325622
**CD14**	APC	HCD 14	Biolegend	325608
**CD16**	BV421	3G8	Biolegend	302038
**CD16**	FITC	3G8	Biolegend	302006
**CD32**	PE	FUN-2	Biolegend	303206
**CD36**	FITC	5-271	Biolegend	336204
**CD36**	PE	5-271	Biolegend	336206
**CD38**	APC	HIT2	Biolegend	303509
**CD64**	APC	10.1	Biolegend	305014
**CD86**	PE	IT2.2	Biolegend	305438
**CD86**	PE/Cy7	IT2.2	Biolegend	305422
**CD137**	PE/Cy7	4B4-1	Biolegend	309818
**CD137**	APC/Cy7	4B4-1	Biolegend	309830
**CD163**	BV421	GHI/61	Biolegend	333612
**CD274**	PE	29E.2A3	Biolegend	329706
**CD274**	APC	29E.2A3	Biolegend	329708
**CD279**	PE	EH12.2H7	Biolegend	329906
**HLA-DR**	APC/Cy7	L243	Biolegend	307618
**IL6**	PE	MQ2-13A5	Biolegend	501107
**pERK**	PE	6B8B69	Biolegend	369505
**TNFa**	AF647	MAb11	Biolegend	502916
**Mfn-2**	-	D2D10	Cell Signaling Technology	9482s
**CD20**	FITC		Miltenyi	120001413
**CD120b**	VioBright FITC	REA520	Miltenyi	130-119-777
**pTyr**	PE	REA374	Miltenyi	130-105-490
**GLUT1**	APC	202915	R&D Systems	FAB1418G
**GLUT2**	PE	199017	R&D Systems	FAB1414P
**GLUT3**	FITC	202017	R&D Systems	FAB1415G

Supplemental Table 3: Primers for mtDNA content

Gene	Forward	Reverse
**MT-TL1**	CACCCAAGAACAGGGTTTGT	TGGCCATGGGTATGTTGTTA
**MT-RNR2**	GCCTTCCCCCGTAAATGATA	TTATGCGATTACCGGGCTCT
**B2M**	TGCTGTCTCCATGTTT-GATGTATCT	TCTCTGCTCCCCACCTCTAAGT

Supplemental Figures

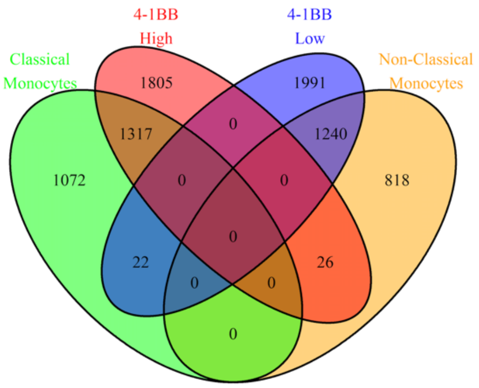

Supplemental Figure 1: Venn diagram comparing the overlap of the differentially expressed genes ($p<0.05$) between CD137^{High}, CD137^{Low} with classical and non-classical monocytes derived from Gene Expression Omnibus Series GSE107011.

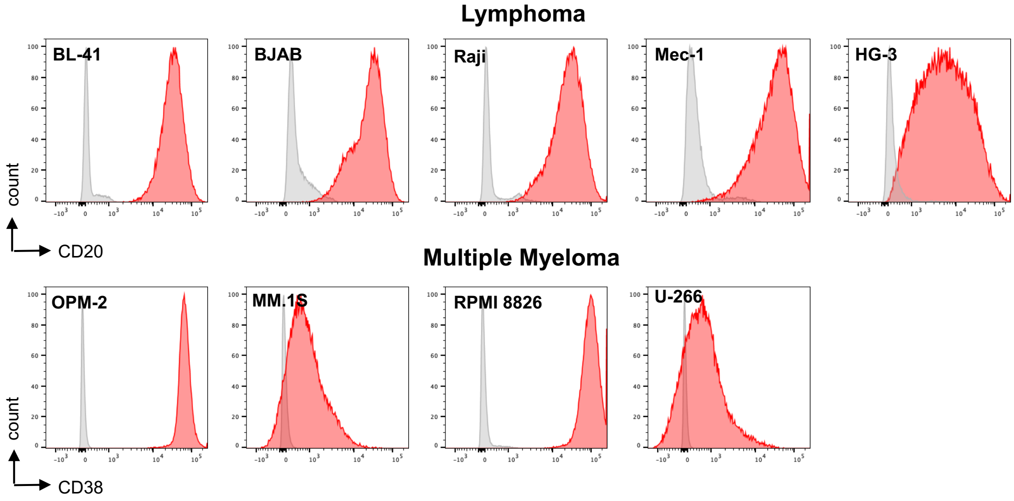

Supplemental Figure 2: Expression of target antigens for therapeutic antibodies on tumor cell lines used for phagocytosis experiments, as determined by FACS (red=stained, gray= unstained). Upper panel depicts representative histograms of CD20 staining on Burkitt lymphoma cell lines BL-41, BJAB, and Raji as well as CLL cell lines Mec-1 and HG-3. Lower panels show CD38 expression on multiple myeloma cell lines OPM-2, MM.1S, RPMI8826, and U-266.

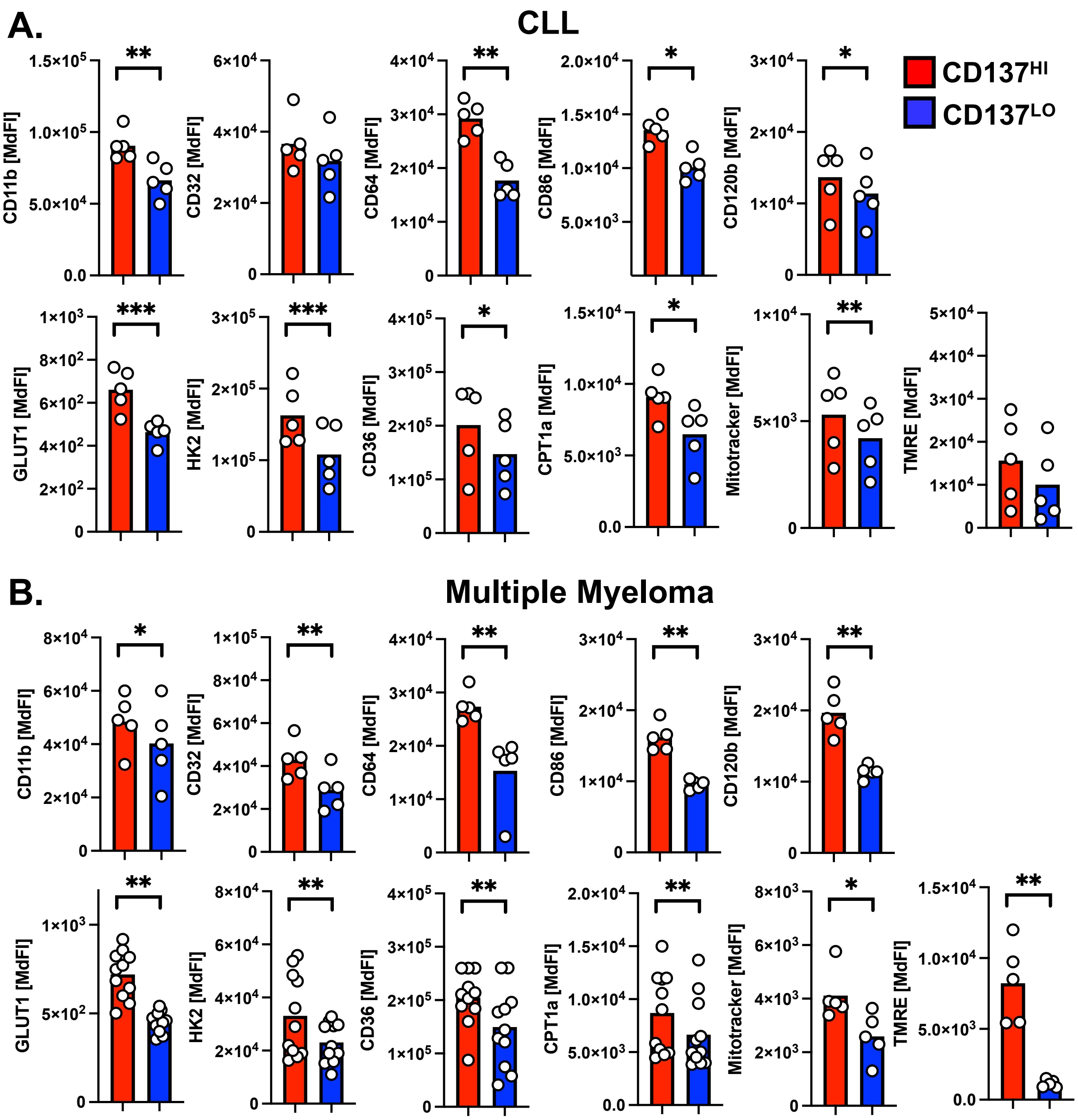

Supplemental Figure 3: Expression of CD11b, CD32, CD64, CD86, CD120b, glucose transporter 1 (GLUT1), hexokinase 2 (HK2), CD36, carnitine palmitoyltransferase I (CPT1) as well as mitochondrial mass (i.e. Mitotracker) and mitochondrial membrane potential (i.e. TMRE) were determined by FACS in CD137^{HI} and CD137^{LO} monocytes from patients with **(A)** chronic lymphocytic leukemia (CLL) or with **(B)** multiple myeloma. Abbreviations: *, p<0.05; **, p<0.01; ***, p<0.001.

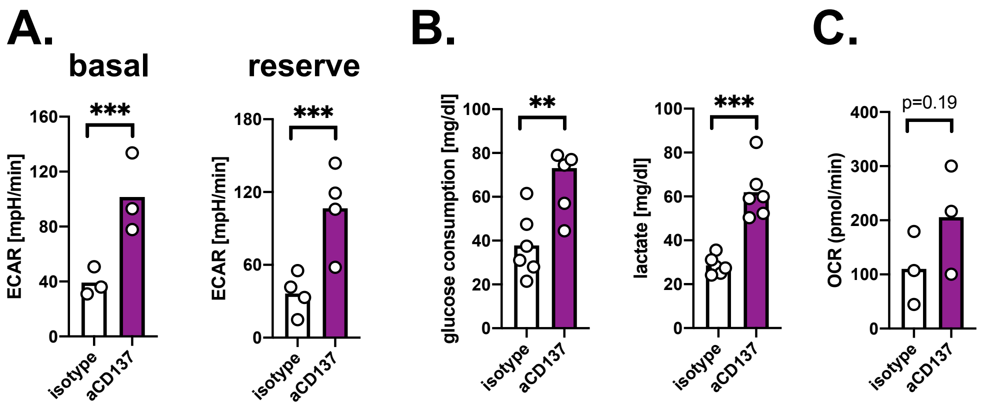

Supplemental Figure 4: (A) Depicted are basal glycolysis after glucose administration of control vs. anti-CD137 mAb-treated monocytes (left panel) and glycolytic reserve capacity after oligomycin administration (right panel). **(B)** Glucose consumption (left panel) and lactate production (right panel) were determined in supernatants of monocytes cultured with either control or anti-CD137 mAb for four days. **(C)** Graph shows basal respiration of control vs. anti-CD137 mAb-treated monocytes. Abbreviations: **, p<0.01; ***, p<0.001.

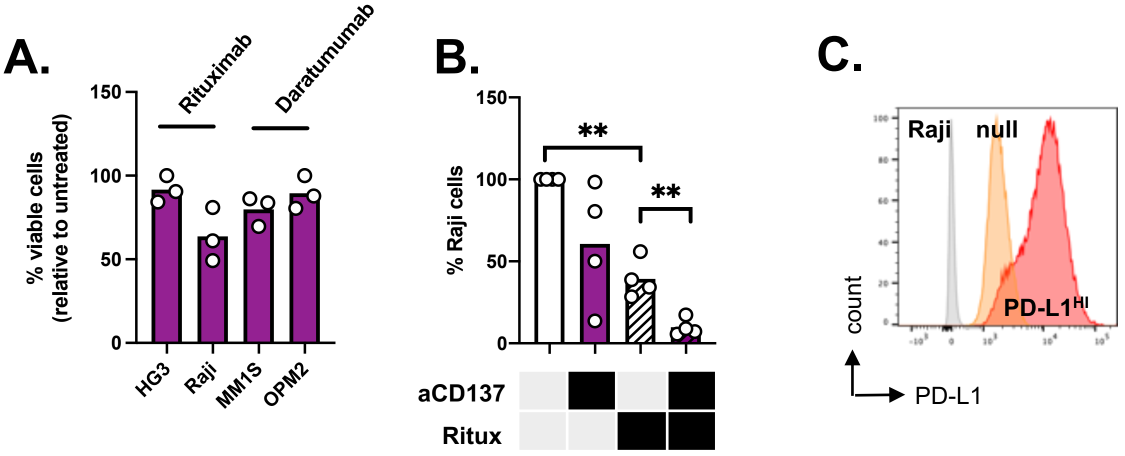

Supplemental Figure 5: (A) Viability of CD20^{+} B-cell-derived Non-Hodgkin Lymphoma cell lines (HG-3 and Raji) and CD38^{+} multiple myeloma cell lines (MM1S and OPM2) in presence of Rituximab and Daratumumab respectively. **(B)** Killing of CPD-stained Raji cells in presence or absence of a therapeutic antibody (Ritux=Rituximab) by monocytes pretreated with either control or anti-CD137 mAb for 3 days. Survival of tumor cells co-cultured with control mAb-treated monocytes without therapeutic antibody are set to 100%. **(C)** Expression of PD-L1 on Raji cell lines as determined by FACS. Red histogram shows PD-L1 expression on Raji PD-

L1 cell line, orange histogram represents PD-L1 expression on Raji null cell line and grey histogram is unstained control. Abbreviations: **, p<0.01.

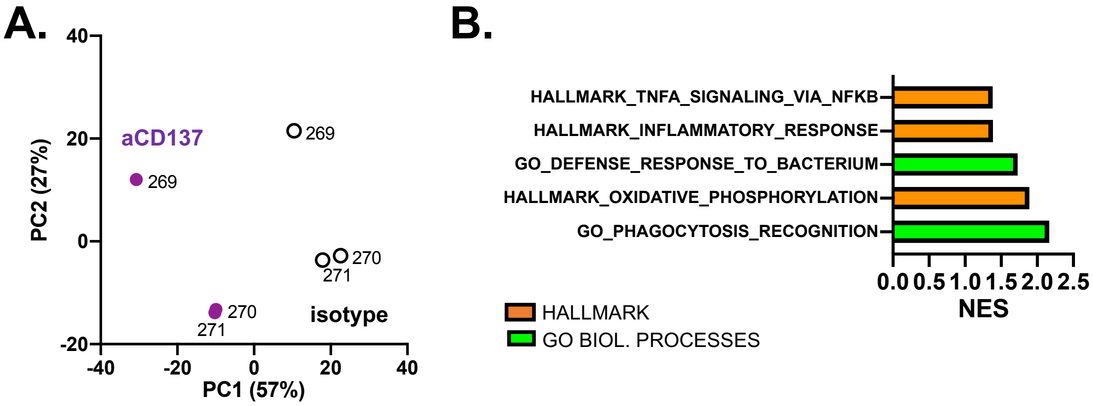

Supplemental Figure 6: **(A)** Principal component analysis of differentially expressed genes between isotype and agonistic anti-CD137 mAb-treated monocytes shows distinct clustering of samples according to treatment. Monocytes were cultured with either control antibody or the anti-CD137 mAb 4B4-1 for four days and subsequently RNA extraction and sequencing were performed (n=3). **(B)** Gene set enrichment analysis of differential gene expression between isotype and anti-CD137 mAb-treated monocytes. Graph shows significantly enriched pathways found in MSigDB hallmark (orange) and biological process ontology (green) gene sets. Size of gene set is annotated to the right. Bar length corresponds to normalized enrichment score (NES).

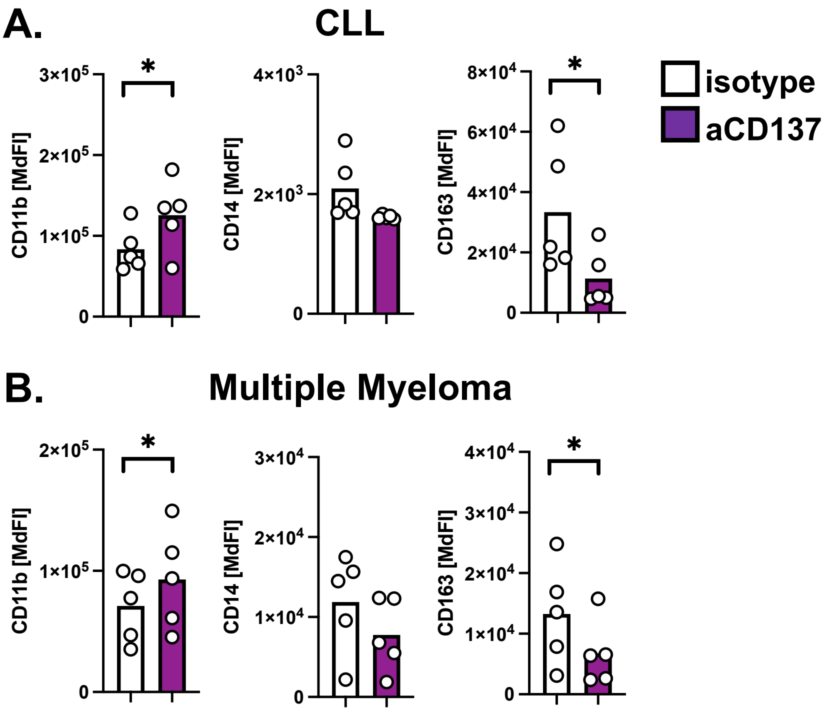

Supplemental Figure 7: Expression of CD11b, CD14, and CD163 was quantified by FACS on monocytes from patients with **(A)** CLL or with **(B)** multiple myeloma treated for four days with a control antibody (i.e. isotype) or an agonistic anti-CD137 mAb. Abbreviations: *, $p<0.05$.

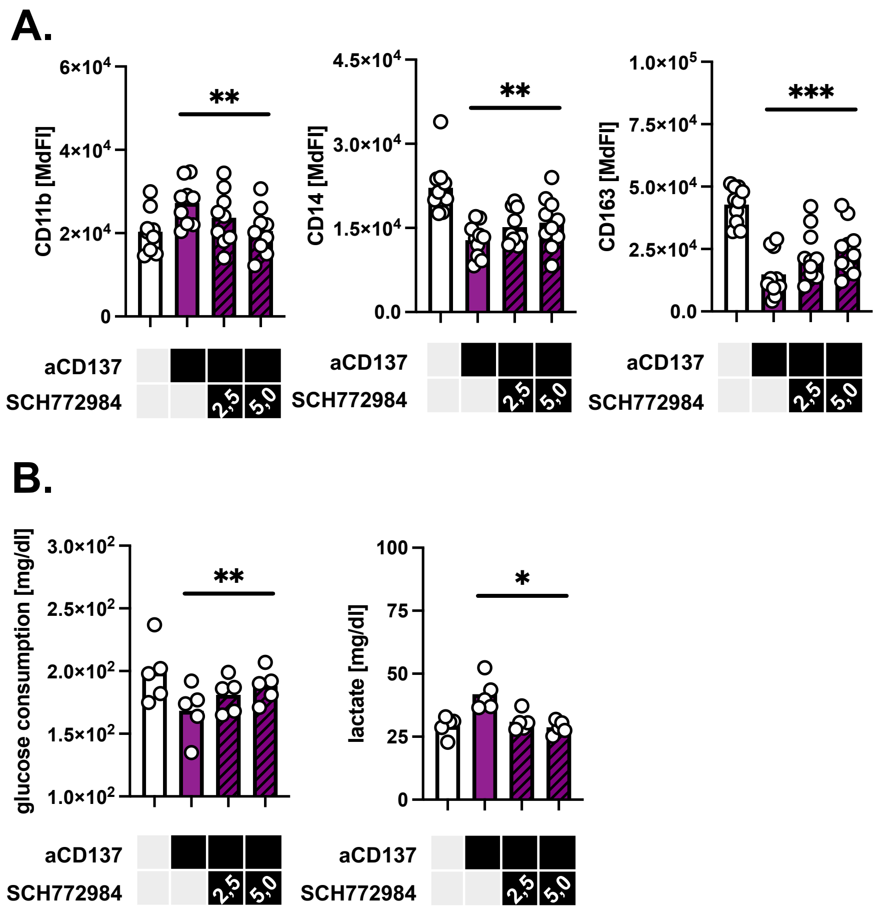

Supplemental Figure 8: **(A)** Expression of CD11b, CD14, and CD163 was determined on monocytes treated with either control antibody, anti-CD137 mAb or a combination of anti-CD137 mAb and the indicated concentrations (in nM) of SCH772984 for four days. (D) Glucose consumption and lactate production were determined in the supernatants of monocytes treated with either control antibody, anti-CD137 mAb or a combination of anti-CD137 mAb and the indicated concentrations (in nM) of SCH772984 for four days. Bars indicate the mean. Abbreviations: *, p<0.05; **, p<0.01.
